# Ankle reconstruction in type II fibular hemimelia

**DOI:** 10.1007/s11751-012-0129-4

**Published:** 2012-03-21

**Authors:** Hazem Mossad El-Tayeby, Amin Abdel Razek Youssef Ahmed

**Affiliations:** 1 Orthopedic Department, Faculty of Medicine, Menoufia University, Shebin El Kom, Egypt; 2 Al-Hadra University Hospital Orthopedic Department, Faculty of Medicine, Alexandria University, Alexandria, Egypt

**Keywords:** Fibular hemimelia, Ankle reconstruction

## Abstract

Ankle reconstruction prior to limb lengthening for was performed in 13 patients with fibular hemimelia with complete radiological absence of the fibula (type II). There were different degrees of absence of metatarsal rays. The hindfoot deformity was a heel valgus in 12 patients and equinovarus in 1 patient. The patients’ ages ranged from 9 to 26 months. Excision of the fibular anlage was performed with lateral subtalar and ankle soft tissue releases to restore the ankle and subtalar joint relationships. In all cases, the fibular anlage ended distally in a cartilaginous lateral malleolar remnant that was fused to the talus in two patients. This fibular remnant was advanced distally and fixed to the tibia with 2 Kirschner wires to recreate an ankle mortise. The period of follow-up ranged from 12 to 38 months. All patients had a stable ankle without tendency to valgus deformity or subluxation. The ankle range of movement was a mean of 27.3° plantarflexion (25–30) and 18° dorsiflexion (15–20). Reconstruction of the ankle in type II fibular hemimelia using advancement of the cartilaginous lateral malleolar remnant has produced encouraging results in the short-term but longer follow-up is needed.

## Introduction

The fibula transfers weight from the knee to the ankle, estimated at 10–16 % by different authors; this reduces to <1 % after a partial resection [1–3]. The lateral malleolus provides ankle stability directly as well as indirectly through the talofibular and calcaneofibular ligaments. Goh et al. [1] conducted a cadaver study to investigate the load-bearing tolerance of the fibula by placing transducers in both the tibia and fibula. They confirmed that fibular load transmission varied according to the position of the ankle. When the ankle was in a neutral position, it transmitted 7.1 % of the load, the maximum load being in dorsiflexion and eversion. When resection was performed proximal to the location of the transducer, load reduction was significant, decreasing to 0.6 %.

Fibular hemimelia is a congenital disorder characterized by partial or complete absence of the fibula. It is the most common longitudinal deficiency of long bones and is a spectrum of anomalies ranging from mild fibular shortening to bilateral involvement with associated defects of the femur, tibia, ankle, and foot. There is a leg-length discrepancy usually with an equinovalgus deformity of the foot; sometimes, there are associated anomalies including a flexion contracture of the knee, femoral shortening, instability of the knee and ankle, and a stiff hindfoot with absent lateral rays [4]. Fibula shortening varies in its extent from minimal shortening to total absence (Achterman–Kalamchi classification) [5]. In fibular hemimelia, the hindfoot lacks lateral support from the fibular malleolus; additionally, the distal tibial epiphysis is wedge shaped and the resulting slope adds to the instability of the hindfoot, which tends to slip off the tibia laterally and dorsally. [6]

Using the Ilizarov technique for the reconstruction of the leg through lengthening and deformity correction [7–10], the surgeon has to confront the risk of subluxation of the ankle when the lateral malleolus is completely absent. Many efforts have been made to prevent this and increase the stability of the ankle joint, but most are either of short-term follow-up or have a high complication rate. We describe an alternative method which used the fibula remnant (anlage) as a buttress for lateral stabilization of the ankle.

## Materials and methods

Thirteen patients with fibular hemimelia type II according to the Achterman–Kalamchi classification were treated by the method described below. There were eight girls and five boys. The youngest patient in this series was 9 months and the eldest 26 months with a mean age of 13 months. The right limb was affected in seven patients (53.8 %) while the left affected in four (30.8 %) and both limbs in two (15.4 %). On the basis of the intraoperative findings, 10 patients had a talocalcaneal coalition. The patients were clinically and radiologically followed up for an average of 18.6 months (12–38 months).

The principle of this method is to use the rudimentary fibular anlage for the reconstruction of the ankle by fixing it to the lateral side of the distal tibia and talus with the aim of fusion and therefore prevent a valgus deformity and lateral subluxation of the ankle.

### Operative steps

1. A lateral soft tissue release of the ankle and Z-Plasty of peroneal tendons and tendo Achillis is performed through a posterolateral incision (Fig. 1a, b)

**Fig. 1:**
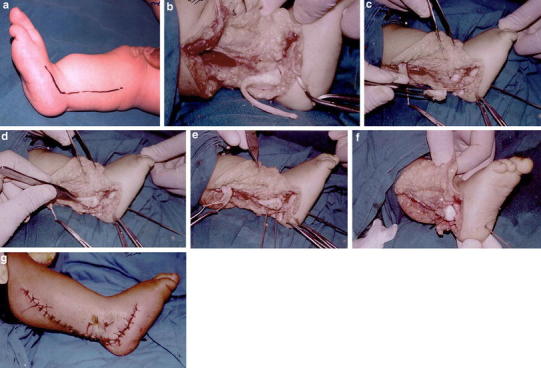
The operative steps: **a** Posterolateral incision. **b** Lateral soft tissue release of the ankle and Z-Plasty of peroneal tendons and tendo Achillis. **c** Excision of the underlying fibular anlage and release of the fibular remnant from its proximal displaced position. **d** Centralization of the ankle by putting the talus under the tibia and fixation by a transcalcaneal wire. **e**, **f** Fixation of the cartilaginous anlage to the tibia and talus using two transverse smooth K-wires. **g** Closure of the wound in layers
2. The underlying fibular anlage and malleolar remnant is excised and released from its proximally displaced position (Fig. 1c).
3. A sharp dissection and division of the talocalcaneal coalition, if present, is performed to reestablish the alignment of the talocalcaneal relationship.
4. The ankle is centralized by placing the talus under the tibia and fixation with a transcalcaneal wire (Fig. 1d).
5. The cartilaginous anlage is made to serve as a lateral support to the ankle by fixing it to the distal tibia and talus using two transverse smooth K-wires; it becomes a ‘lateral malleolus’ buttressing the talus (Fig. 1e, 1f).
6. The wound is closed in layers and an above knee plaster cast applied (Fig. 1g).

### Postoperative management

The sutures were removed after 2 weeks and the cast after 6–8 weeks.

## Results

**Fig. 2:**
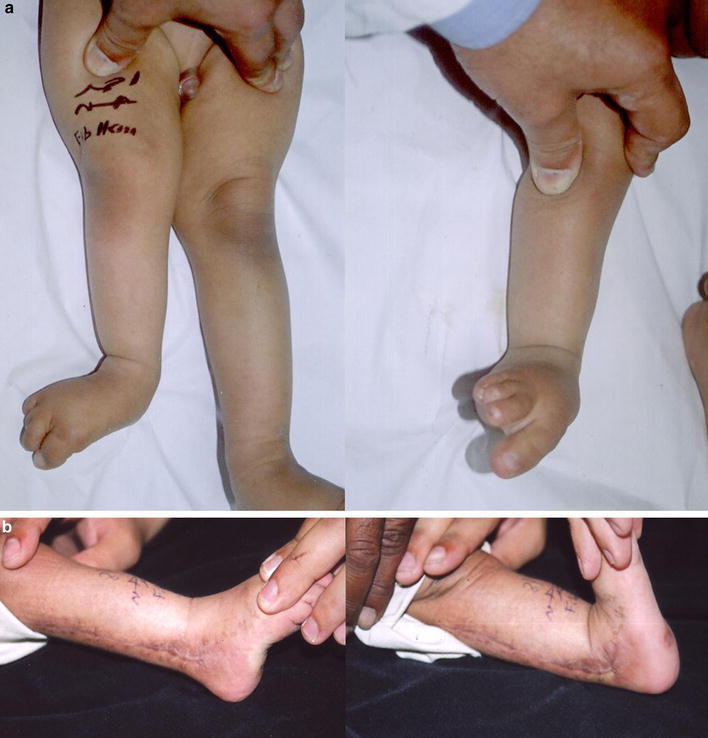
Case example: 14-month-old boy with right fibular hemimelia. **a** Preoperative photographs of the patient. **b** Photographs of the patient after 18 months of follow-up with regained ankle range of motion

The period of follow-up ranged from 12 to 38 months. The patients were found to have a stable ankle without tendency to valgus deformity or subluxation of the ankle with a range of ankle movement ranging from a mean of 27.3° (25–30) plantarflexion and 18° (15–20) dorsiflexion (Fig. 2). The knee retained full range of motion. The presence of a talocalcaneal did not influence the final range of ankle dorsiflexion and plantarflexion.

Limb length discrepancy ranged from 3 to 6.5 cm with a brace (AFO) applied for all patients to compensate for shortening until the age of lengthening at 4–5 years. X-ray follow-up showed good alignment in all cases but without any evidence of ossification of the fibular anlage.

## Discussion

The absence of the lateral malleolus in fibular hemimelia causes subluxation and valgus deformity of the ankle joint due to lack of the lateral support at the ankle. Excision of the fibular anlage and centralization of the ankle corrects the valgus deformity but does not prevent the gradual subluxation and valgus deformity that usually recurs with growth and with tibial lengthening.

Attempts have been described to increase ankle stability in patients with fibula hemimelia. The osteotomy described by Ulrich Exner which altered the convexity of the distal tibia to a concave shape showed promising short-term results [11]. Reconstruction of the lateral malleolus using free bone grafts has led to recurrence. Use of epiphyseal cartilage or a vascularized fibular head has been described, but complications were also noted including those affecting the normal (donor) limb and those associated with microvascular surgery [12].

Grucca described transepiphyseal division of the plafond and a proximal and medial translation of the medial tibial fragment with the talus accompanying it, but many complications, notably damage to the physeal growth and bony bar formation, growth retardation, and ankle arthrosis, were reported [13, 14].

Michael Weber described a lateral malleolus reconstruction in a 5.5-year-old boy with type II fibular aplasia using a triangular iliac crest transplant to the lateral distal tibia with the apophysis as a growing portion and the gluteal fascia as a lateral ligament. He reported a good result after 2.5-year follow-up with ankle stability and a good range of movement [15].

Finally, as a salvage procedure, arthrodesis of the ankle has been recommended by several authors.

Using the fibular anlage for the reconstruction of the lateral malleolus solves the problem of instability and prevents subluxation of the ankle and progressive valgus deformity of the ankle. This is illustrated in this series of short-term follow-up. All patients were found to have a stable ankle joint without any subluxation or valgus deformity and a good range of motion. This suggested that the transplanted anlage had not fused and acted only as an extra-articular buttress to the lateral side of the ankle. We did not identify ossification of the cartilaginous anlage in the period of follow-up (12–38 months). In those patients who underwent an MRI scan (6 patients), we were not able to identify the anlage remnant. Longer follow-up is needed to ensure that the stability of the ankle with growth of the child or during leg lengthening is maintained.

## Conclusion

We can reservedly recommend the anlage remnant of fibula as a potential donor material for lateral malleolar creation in ankle reconstruction for type II fibular hemimelia. It acts as a buttress to the talus and prevents the recurrence of valgus deformity.
